# Serum TRSUT Titer ≥1:16 Is a Predictor for Neurosyphilis Among HIV-Infected Patients With Concurrent Syphilis and No Neurological Symptoms

**DOI:** 10.1097/MD.0000000000002023

**Published:** 2015-11-13

**Authors:** Jian-Jun Sun, Zhen-Yan Wang, Jia-Yin Shen, Yin-Zhong Shen, Li Liu, Jiang-Rong Wang, Ren-Fang Zhang, Hong-Zhou Lu

**Affiliations:** From the Department of Infectious Disease, Shanghai Public Health Clinical Center, Fudan University (J-JS, Z-YW, J-YS, Y-ZS, LL, J-RW, R-FZ, H-ZL); Department of Infectious Disease, Huashan Hospital Affiliated to Fudan University (H-ZL); and Department of Internal Medicine, Shanghai Medical College, Fudan University, Shanghai, China (H-ZL).

## Abstract

Investigating the predictors for lumbar puncture to diagnose the asymptomatic neurosyphilis among HIV and syphilis co-infected patients in Shanghai, China.

Respectively, screening the medical records from August 1, 2009 to June 30, 2015. Those HIV-infected patients with concurrent syphilis who had received lumbar puncture were selected and their clinical and demographic data were recorded. Participants comprised symptomatic and asymptomatic patients. The latter ones could be further divided into 3 groups: late syphilis, early syphilis with anti-syphilis treatment failure, and early syphilis with serum toludine red unheated serum test (TRUST) ≥1:32. Both syphilis stage and anti-syphilis treatment effect were defined by common criteria, and syphilis of unknown duration was considered as late syphilis. Asymptomatic neurosyphilis was defined as neurosyphilis without neurological symptoms such as headache, cognitive dysfunction, motor deficits, auditory or ophthalmic abnormalities, and stroke. Neurosyphilis was defined as reactive cerebrospinal fluid (CSF) TRUST and/or CSF white blood cell >20 cells/μL without other reasons. Mann–Whitney test and Fisher's exact test were used for analyzing the difference between neurosyphilis and non-neurosyphilis group. Logistic regression test was performed to analyze the risk factors for neurosyphilis.

In total, 170 participants were collected, and the rate of neurosyphilis was 32.35%. Among all the 105 participants without neurological symptoms, 80 patients were with late syphilis and 25 were with early syphilis. Among the early syphilis patients, 23 had a TRUST ≥1:32 and the other 2 experienced an anti-syphilis treatment failure. The differences of clinical and demographic variables between neurosyphilis and non-neurosyphilis group were not statistically significant except the serum TRUST titer (*P* < 0.01). From HIV/syphilis co-infected patients with or without neurological symptom, those who had neurological symptoms, CD4 <350 per μL and serological TRUST titer ≥1:16 were 4.9-fold (95% confidence interval [CI]: 2.37–10.31), 4.3-fold (95% CI: 1.17–15.78), and 4.1-fold (95% CI: 1.58–10.76), respectively, more likely to be diagnosed with neurosyphilis. Asymptomatic patients whose serum TRUST titer ≥1:16 were 8.48-fold (95% CI: 1.08–66.63) more likely to have asymptomatic neurosyphilis.

Among asymptomatic HIV-infected patients with late syphilis or early syphilis experienced an anti-syphilis treatment failure, those who have a serum TRUST titer ≥1:16 are suggested to perform lumbar puncture in order to avoid delayed diagnosis and the occurrence of severe sequelae of syphilis.

## Abstract 2

Supplemental Digital Content is available in the text

## INTRODUCTION

Syphilis is a systemic disease caused by *Treponema pallidum*, and this agent can invade the central nervous system and result in neurosyphilis which could occur at any stage of syphilis.^1^ Syphilis shares the transmission route with HIV, and it can facilitate HIV infection.^2^ Both syphilis and HIV infection have been becoming a serious public health burden worldwide for many years.^3^ The findings from some studies^4–6^ confirmed that the prevalence of syphilis was 14.32% to 20.34% among men who have sex with men while the syphilis prevalence among HIV-infected patients was 19.8%. The research^7^ from Shanghai Skin Disease Hospital found the rate of neurosyphilis was 23.1% among syphilis patients without HIV infection. Alteration of the immunity during HIV infection appears to enhance the persistence of *T pallidum* in the central nervous system, which makes neurosyphilis occur more frequently, progress more rapidly, and present with atypical signs.^8,9^ Among HIV-infected patients, neurosyphilis shows a lower age of onset and a higher proportion of early forms.^10^ Without timely diagnosis and effective treatment, severe sequelae of syphilis could occur. However, the diagnosis of the central nervous system involvement is difficult for patients without typical clinical manifestations. So far, there is no simple and single definitive laboratory test for neurosyphilis diagnosis. Therefore, the common definition for diagnosis of neurosyphilis depends on a combination of cerebrospinal fluid (CSF) tests (CSF cell count or protein and a reactive CSF-venereal disease research laboratory (VDRL)).^1^ It is well known that the CSF-VDRL analysis is a recommended method for the laboratory diagnosis of neurosyphilis. However, the procedure of CSF-VDRL is not only complicated but also time-consuming,^11^ for which the reagents need to be freshly prepared and used within a short time.^7^ Furthermore, in China, there are no commercial VDRL reagents that have been approved by the China Food and Drug Administration for CSF-VDRL examination, while multiple commercial toludine red unheated serum test (TRUST) reagents are available for use in the laboratory diagnosis of syphilis.^7,12^ According to the reports in the published studies,^7,13,14^ the CSF-TRUST may be used as an alternative for laboratory diagnosis of neurosyphilis in clinical settings when CSF-VDRL unavailable. So in the daily analysis of CSF samples, many clinical laboratories take TRUST as a substitute for the VDRL test. Since the optimal time point to perform the lumbar puncture among HIV-infected patients with concurrent syphilis is still controversial,^1,15,16^ it is also hard to decide when to perform CSF test for the diagnosis of neurosyphilis. For those HIV-infected patients with syphilis who have neurological symptoms, lumbar puncture is necessary not only for the diagnosis of neurosyphilis but also for differential diagnosis such as bacterial meningitis, tuberculous meningitis, and cryptococcal meningitis. As a matter of fact, given the inconvenience of lumbar puncture, the appropriate time for CSF test among those HIV and syphilis co-infected patients without neurological symptoms or signs are really crucial but hard to decide. Many researchers have made analysis on when to perform lumbar puncture among patients with HIV and syphilis co-infection including both symptomatic and asymptomatic syphilis^8,10,15^; however, studies focused on asymptomatic neurosyphilis was limited and the sample size was small.^17^

As far as China is concerned, given the limitations of VDRL test, the CSF examinations were mainly performed by TRUST or rapid plasma regain (RPR) which is a modification of VDRL. However, there is no study based on the TRUST results to evaluate the predictors for neurosyphilis, especially asymptomatic neurosyphilis. So the objective of our study is to analyze the risk factors for the asymptomatic neurosyphilis among HIV-infected patients with concurrent syphilis in Shanghai China.

## METHODS

### Ethics Statement

This research protocols were approved by the Shanghai Public Health Clinical Center Ethics Committee. Given the data were analyzed retrospectively and anonymously, the committee decided to waive the need for written informed consent from the participants in this study.

### Study Design

Eligible participants selected from August 1, 2009 to June 30, 2015 in Shanghai Public Health Clinical Center Affiliated to Fudan University which is a tertiary teaching hospital. The inclusive criteria: HIV antibody positive (by western blot); age more than 18 years old; both serum *T pallidum* particle agglutination (TPPA) and TRUST were positive; having CSF test results (TRUST titer and white blood cell [WBC] count). Exclusive criteria: Patients with neurological symptoms taken lumbar puncture for reasons such as bacterial meningitis, tuberculous meningitis, and cryptococcal meningitis, but not for neurosyphilis diagnosis. And we, respectively, divided these participants into 2 groups: one was asymptomatic when the lumbar puncture was performed; the other one was symptomatic when lumbar puncture was given. The neurological symptoms include^1^: headache, cognitive dysfunction, motor or sensory deficits, auditory or ophthalmic abnormalities, and symptoms or signs of meningitis or stroke. For the asymptomatic patients with HIV/syphilis co-infection given lumbar puncture examination comprising: Patients with concurrent syphilis and HIV infection who have late syphilis (including late latent syphilis and syphilis of unknown duration), regardless of CD4 counts or TRUST titers; patients with syphilis and HIV infection who has serum TRUST titer ≥1:32, irrespective of CD4 counts or syphilis stage; patients with inadequate serologic response to recommended syphilis treatment in China (benzathine penicillin G, 2.4 M, intramuscular injection QW ∗ 3 weeks). The definitions of inadequate response are: without a ≥4-fold decrease of the TRUST titer 12 months after receipt of appropriate therapy for syphilis or a ≥4-fold increase of the TRUST titer more than 30 days after receipt of recommended syphilis therapy.^1^ The syphilis stage was determined according to latest America CDC guidelines^1^; patients with syphilis of unknown duration were included in the group of late syphilis. Results of serum TPPA/TRUST results and peripheral blood CD4 count were obtained from medical records. All of these test results were performed within 30 days of the lumbar puncture time. Serum TRUST and TPPA tests and CSF WBC count and CSF TPPA, TRUST tests were performed using standard methods at the central laboratory of our hospital which had been qualified by the China Ministry of Health. Neurosyphilis was defined as a reactive CSF-TRUST test result and/or CSF WBC count >20 cells/μL without other reasons, which was above the mild level of pleocytosis usually seen with HIV alone.^1,16^ Asymptomatic neurosyphilis was defined as neurosyphilis without neurological symptoms which were listed above.

### Analysis and Statistics

Data analysis was conducted by IBM SPSS version 19.0 (IBM SPSS, Inc., Armonk, NY). Continuous variables were described using median and interquartile range (IQR) while categorical variables were described by numbers and percentages. The Fisher's exact test was used for categorical variables and Mann–Whitney test for continuous variables (chi-squared test and *t* test were not suitable for these data according to the principle of statistics). We used logistic regression test to analyze the risk factors for neurosyphilis. The confounding factors included: patient's age, level of CD4 cell counts, having symptoms when performing lumbar puncture, TRUST titer and syphilis stage. All hypothesis testing was 2-sided, with a level of α = 0.05. The cut-off values we used for logistic regression test were: TRUST titer of 1:16, 1:32, 1:64; CD4 level of 250 per μL, 350 per μL. All of the clinical data underlying these findings are listed in the Supplementary Material File (S1 File).

## RESULTS

**FIGURE 1:**
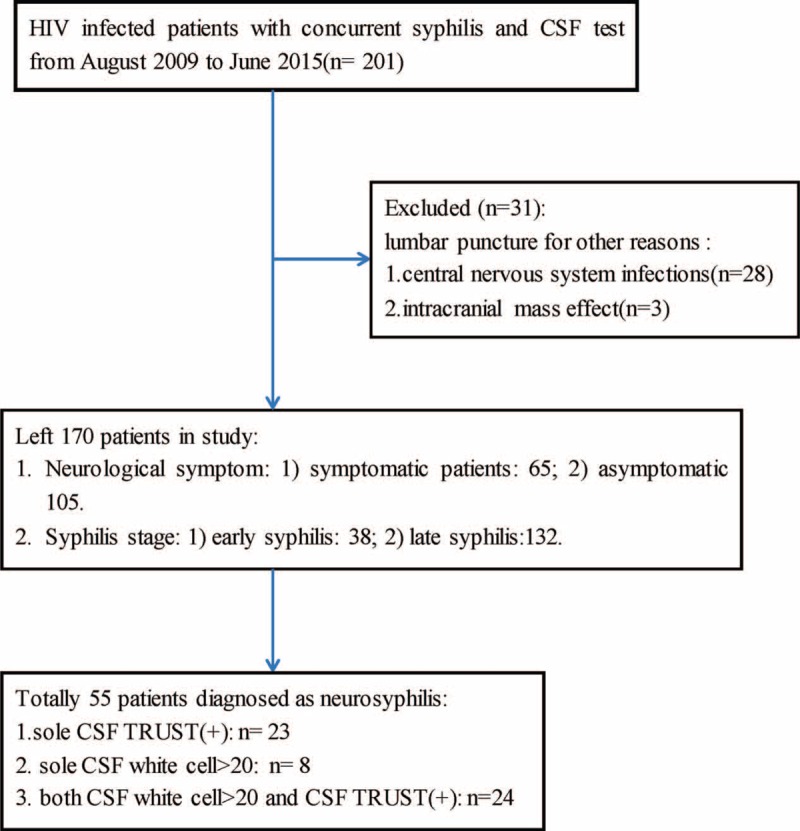
Flow chart of participants’ selection. TRUST = toludine red unheated serum test, CSF = cerebrospinal fluid, late syphilis = including late latent syphilis and syphilis with unknown duration.

Among the 201 patients who fulfilled the inclusive criteria during the period from August 1, 2009 to June 30, 2015, 31 patients were ruled out according to the exclusive criteria. Totally 55 patients out 170 eligible participants were diagnosed as having neurosyphilis, therefore the rate of neurosyphilis was 32.35% (Figure 1).

Among the 170 patients, 5 participants were female and 165 were male. The age range of neurosyphilic patients was 21 to 62 years and that of non-neurosyphilic patients was 18 to 65 years. CD4 cell counts of patients with neurosyphilis and with non-neurosyphilis ranged from 1 to 497 per μL and 1 to 940 per μL, respectively. And for the titers of serum TRUST, the range was 1:1 to 1:1024 for patients with neurosyphilis and 1:1 to 1:512 for those with non-neurosyphilis. For the patients with neurosyphilis defined by the diagnostic criteria of CSF WBC >20 per μL, the median count of WBC was 49 per μL (range 25–145 per μL). Among the asymptomatic patients with HIV/syphilis co-infection, 80 patients were found to have late syphilis and 25 patients had early syphilis. For those 25 early syphilis patients, 23 had a serum TRUST titer ≥1:32 (1:32–1:512) and the remaining 2 patients experienced an anti-syphilis treatment failure. Among all the 65 HIV/syphilis co-infected patients with neurological symptoms, 17 (26.2%) had headache, 5 (7.6%) cognitive dysfunction, 12 (18.5%) motor or sensory deficits, 2 (3.1%) auditory abnormalities, 26 (40%) ophthalmic abnormalities, and 3 (4.6%) stroke.

The differences of gender, age, syphilis stage, and antiretroviral therapy (ART) duration between neurosyphilis group and non-neurosyphilis group were not statistically significant, so did the CD4 cell counts (Table 1). However, the titers of serological TRUST were significantly different (*P* < 0.01).

**TABLE 1:**
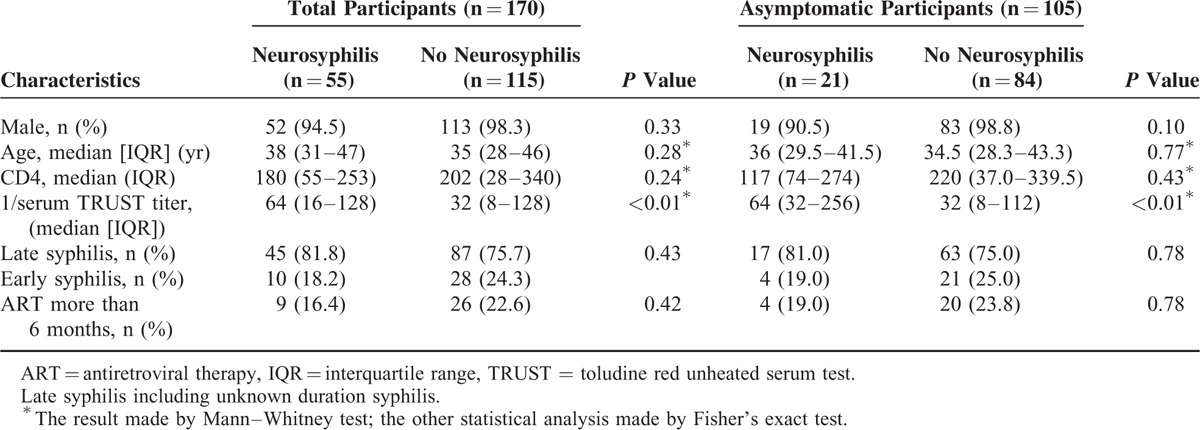
Characteristics of Total Participants or Only Asymptomatic Participants With or Without Neurosyphilis

For the HIV-infected patients with concurrent syphilis, those who had neurological symptoms when performing lumbar puncture, CD4 cell counts <350 per μL, and with serological TRUST titer ≥16 was 4.9-, 4.3-, and 4.1-fold, respectively, more likely to be diagnosed as having neurosyphilis (Table 2).

**TABLE 2:**
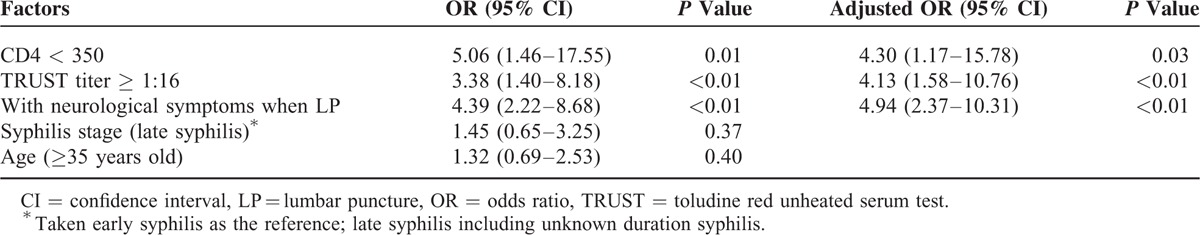
Risk Factors for Neurosyphilis Among 170 HIV/Syphilis Co-Infected Patients With or Without Neurological Symptoms

For HIV/syphilis co-infected patients without neurological symptoms, those who had serum TRUST titer ≥1:16 was 8.48-fold (95% confidence interval [CI]: 1.08–66.63, *P* = 0.04) more likely to have asymptomatic neurosyphilis. However, other factors, such as the level of CD4 cell count, syphilis stage, and the duration of ART, were not predictors for having asymptomatic neurosyphilis.

## DISCUSSION

To our knowledge, this is the first time that serum TRUST was used to analyze the risk factors for asymptomatic neurosyphilis among HIV/syphilis co-infected patients. The rate of neurosyphilis in our study was 32.35%, and it is higher than that from other studies.^8,17^ Between patients with and without neurosyphilis, the median serum TRUST titer was significantly different. For all the HIV/syphilis co-infected patients with or without neurological symptoms, the risk factors of neurosyphilis were having neurological symptoms, CD4 <350 per μL, serological TRUST titer ≥1:16. This result was consistent with the previous researches^8,18^; however, the titer value was different from that of their findings. Of note, the participants’ composition of our study was similar with the other studies.^8,18^ Given that our neurosyphilis rate and predictors were similar to those from the previous studies, it implicated that our study design and CSF test method were reasonable and reliable. The TRUST was modified from RPR,^7^ it might have different levels of quantitative titers for the same serum sample just like the differences of titers between VDRL and RPR.^1^ Actually, the serological TRUST titer ≥1:16 was also pointed out by Jiang et al^13^ as an indicator for neurosyphilis in their study. Thus, for HIV/syphilis co-infected patients with or without neurological symptoms, CD4 <350 per μL, serological TRUST titer ≥1:16, and neurological symptoms such as hearing loss, vision deficiency, and headache were predictors for performing CSF test for the diagnosis of neurosyphilis.

As mentioned earlier, it is not a problem to decide the time for lumbar puncture to diagnose neurosyphilis among HIV/syphilis patients with neurological symptoms. And there is no doubt that doctors will perform lumbar puncture to find out the illness for those patients with neurological symptoms no matter what their serum titers are or at which syphilis stage they are. After all, there are so many kinds of central nervous system infections among HIV-infected patients such as bacterial meningitis, tuberculous meningitis, and cryptococcal meningitis.^19,20^ So the real challenge is to decide whether or when to perform the lumbar puncture among HIV/syphilis-infected patients without neurological symptoms.

After we excluded the patients with neurological symptoms, 105 participants were left. According to both univariant and multivariant logistic regressions, we found that CD4 cell count was not a predictor for the asymptomatic neurosyphilis and only the serological TRUST titer was associated with the diagnosis of asymptomatic syphilis. Patients with serum TRUST titer ≥1:16 were 8.48 times more likely to be diagnosed as having asymptomatic neurosyphilis. Thus, the level of CD4 cell count was no long considered a predictor for asymptomatic neurosyphilis in our analysis, though this was different from the previous reports.^8,15,18^ The reasons might include: first, the composition and size of our asymptomatic participants sample was different from those who have been reported; second, the repetitive infection of syphilis was produced by frequent high-risk behavior among asymptomatic patients. We assumed that the repetitive high-risk behavior resulted in syphilis re-infection frequently, and actually it is common among the HIV patients.^6,21^ It is well known that patients with any levels of CD4 cell count could get infected by *T pallidum*, presumably, the CD4 cell counts was not related to the diagnosis of syphilis or neurosyphilis.^22^ However, we were unable to explain the finding that CD4 <350 per μL was a predictor for neurosyphilis among all the co-infected patients with or without neurological symptoms. Therefore, we thought that the different composition of sample was the main reason for this discrepancy. So for the asymptomatic HIV/syphilis co-infected patients with late syphilis or early syphilis experienced antisyphilis treatment failure, only the serum TRUST titer ≥1:16 was a predictor for lumbar puncture examination.

There are some limitations need to be noted in this study. First, there are no follow-up data to evaluate the effect of therapy for patients with confirmed neurosyphilis. Admittedly, this kind of assessment is important and needs to be done in the future with a prospective cohort. Second, as a retrospective analysis, the quality of this study is limited by the incompleteness of the data such as HIV load and its transmission route. However, the correlation between HIV and CSF WBC counts is not statistically significant,^18^ and HIV load would not influence the diagnosis of neurosyphilis. Furthermore, we had no detailed data to classify the participants as experienced syphilis treatment and syphilis without treatment (in our study, only 2 patients were regarded as treatment-failure and it was unconventional). As a matter of fact, given the abuse of antibiotics in China^23,24^ many patients should be considered to have received antisyphilis treatment (such as penicillin, ceftriaxone, erythromycin, or doxycycline) without clear medical records or determined recall in history, and we did not take into account the difference between patients with and without antibiotics treatment for syphilis. However, published literature implicates that this factor is also independent of the risk of neurosyphilis.^18^ Last but not least, in view of the sample we selected, the findings of our study should be used prudently, and it should not be applied to all the HIV/syphilis co-infected patients.

## CONCLUSION

The rate of neurosyphilis among HIV/syphilis co-infected patients in Shanghai is a little higher than that of other studies. Among asymptomatic HIV/syphilis co-infected patients with late syphilis or early syphilis experienced antisyphilis treatment failure, those whose serum TRUST titer ≥1:16 are suggested to perform lumbar puncture in order to get timely diagnosis and avoid severe sequelae of syphilis.

## Supplementary Material

Supplemental Digital Content
